# *Lactiplantibacillus plantarum* ST-III-fermented milk improves autistic-like behaviors in valproic acid-induced autism spectrum disorder mice by altering gut microbiota

**DOI:** 10.3389/fnut.2022.1005308

**Published:** 2022-11-24

**Authors:** Yilin Zhang, Min Guo, Hongfa Zhang, Yuezhu Wang, Ruiying Li, Zhenmin Liu, Huajun Zheng, Chunping You

**Affiliations:** ^1^State Key Laboratory of Dairy Biotechnology, Shanghai Engineering Research Center of Dairy Biotechnology, Dairy Research Institute, Bright Dairy & Food Co., Ltd., Shanghai, China; ^2^NHC Key Lab of Reproduction Regulation (Shanghai Institute for Biomedical and Pharmaceutical Technologies), Fudan University, Shanghai, China; ^3^Shanghai-MOST Key Laboratory of Health and Disease Genomics, Chinese National Human Genome Center at Shanghai and Shanghai Institute for Biomedical and Pharmaceutical Technologies, Shanghai, China

**Keywords:** *Lactiplantibacillus plantarum* ST-III, fermented milk, autism spectrum disorder, dietary intake changes, gut microbiota, Gut-Microbiota-Brain Axis

## Abstract

**Introduction:**

Autism spectrum disorder (ASD) is a serious neurodevelopmental disorder with a rising incidence. More and more studies have shown that abnormal microbiota composition may aggravate the behavioral symptoms and biological signs of ASD, and interventions of probiotics and diet have emerged as a potential improvement measure.

**Methods:**

*Lactiplantibacillus plantarum* ST-III-fermented milk was applied as an oral intervention in a valproic acid (VPA)-induced ASD mice model, and the effect of probiotic intake on autistic-related behaviors and gut microbiota composition was evaluated by behavioral tests and 16S rRNA gene sequencing.

**Results:**

Gender specificity was shown in VPA-induced behavioral abnormalities in a mouse model, and *L. plantarum* ST-III-fermented milk was effective in ameliorating the impaired social interaction in male ASD mouse models, but not for the anxiety behavior exhibited by female ASD mouse models. Meanwhile, dietary changes were found to be the main cause of the altered gut microbiota in mice, and additional intake of *L. plantarum* ST-III-fermented milk seemed to improve autistic-like behaviors in male ASD mouse models by modulating specific gut microbes.

**Discussion:**

These findings suggest that *L. plantarum* ST-III-fermented milk may play a beneficial role in improving the behavioral symptoms of ASD and is expected to be one of the candidate functional foods for ASD.

## Introduction

Autism spectrum disorder (ASD) is a serious neurodevelopmental disorder frequently occurring in infants and young children. It is characterized by communication and social disorders, restricted interests and repetitive stereotyped movements (1). ASD has been reported to be increasing in prevalence rate worldwide (2). According to the latest data released by the US Centers for Control and Prevention, the prevalence of ASD increased from 1:68 in 2016 to 1:59 in 2018 (a 15% increase in prevalence) (3). China had an average prevalence rate of 0.70%, with approximately 1 in 143 children suffering from ASD (4). Consistent with the results reported in other literature (5), the prevalence ratio of males to females in Chinese children with ASD was 4.3:1 (0.95% in boys and 0.3% in girls) (4). ASD is currently thought to be the result of the interaction of both genetic and environmental risk factors (6), with prenatal or postnatal exposure to chemicals and drugs, air pollution, stress, uterine cavity infections and dietary factors all increasing the risk of developing ASD (7). For example, previous studies have shown that taking valproic acid (VPA, a drug for epilepsy and mood swings) during pregnancy greatly increases the risk of ASD in offspring (8, 9).

Among the multiple comorbidities of ASD, variable levels of gastrointestinal dysfunctions are frequently reported, together with changes in the intestinal microbiota (10, 11). Children with ASD are 3.5 times more likely to develop gastrointestinal disorders (12), including chronic constipation, abdominal pain, diarrhea, gastroesophageal reflux, gastrointestinal inflammation and enteric nervous system abnormalities (13). They are also more prone to problems such as irritability, anxiety and social withdrawal, but experience a reduction in the severity of their illness after their gastrointestinal symptoms are improved (14). Studies have found that the gut microbiota of children with ASD significantly differ in composition compared to healthy controls and have a certain correlation with gastrointestinal (GI) symptoms and ASD developing (15). Sharon et al. (16) transplanted gut microbiota from human donors with ASD or typically developing (TD) controls into germ-free mice and found that mice colonized with ASD microbiomes exhibited hallmark autistic-like behaviors, such as repetitive behaviors and social avoidant. Several meta-analyses have reported reductions in *Bifidobacterium* and increase in *Faecalibacterium* and *Clostridium* in children with ASD (17). Another study has found that compared with control group, children with ASD have lower gut microbiota diversity and higher levels of *Bacteroidetes*, *Lactobacillus*, *Clostridium*, *Desulfovibrio*, *Caloramator*, and *Sarcina* (18). These associations have inspired research on the intertwined relationships among gut microbiota, ASD and behavior (19). However, the exact microbial composition associated with the occurrence of ASD has not been determined, with conflicting results at the phylum, genus and species levels and in diversity studies (20). Furthermore, it is still unclear whether microbiota alterations appear as a consequence of ASD or are involved in its onset. Despite discrepancies and controversies between studies remain, more and more research suggests that abnormal microbiota composition may aggravate the behavioral symptoms and biological signs of ASD (21).

Autism spectrum disorder is placing a heavy burden on both the society and families. Unfortunately, there are currently no specific therapies for such disability. Rather than finding a cure for ASD patients, current research on autism has focused on how to relieve symptoms (22). Notably, modulation of the gut microbiota in patients with ASD through interventions of probiotics and diet has emerged as a potential improvement measure. Host diet is one of the most effective regulators of the gut microbiome (23). Changes in dietary macronutrients (including carbohydrates, proteins, fats, vitamins, etc.) lead to rapid and comprehensive changes in the composition of the gastrointestinal microbiota (24). Moreover, some studies have found that interfering with one or more specific probiotics could improve the integrity of gut barrier in ASD mouse model, restore the abundance of specific microbes, and reverse the behavioral abnormalities associated with ASD (25).

*Lactiplantibacillus plantarum* (*L. plantarum*) is a gram-positive lactic acid bacteria species (26). *L. plantarum* can survive under different environmental conditions and is one of the few probiotics that can colonize the gastrointestinal mucosa of the human body (27). It has also been shown to be an important probiotic strain with multiple beneficial effects on intestinal health, metabolic disorders and brain health (28). *L. plantarum* ST-III is a probiotic strain originally isolated from a Chinese traditional pickle (29), which can effectively regulate human cholesterol levels (30). In an *in vitro* study, Yan et al. (31) found that *L. plantarum* ST-III could reduce the overall number of *Enterobacteria* and *Bacteroidetes* and increase the abundance of *Lactobacillus* and certain *Bifidobacterium* species in infant gut microbiota. Zang et al. (32) found that long-term feeding of *L. plantarum* reduced anxiety-like behaviors in zebrafish caused by triclosan exposure and improved their motor activity and learning ability. It has also been found in clinical experiments that single-strain intervention of *L. plantarum* can improve autism-related behaviors such as destructive behavior, anxiety, and communication disorder (33). However, there are few studies have reported on the effects of *L. plantarum* fermented milk on autism-like behaviors. In the published studies, researchers only used *L. plantarum* probiotic capsules or bacterial pellet for intervention experiments (32, 34), which may miss the efficacy of probiotic fermentation metabolites and dietary nutrients in regulating gut microbiota. That’s because many strains of the *L. plantarum* (including *L. plantarum* ST-III, etc.) are difficult to proliferate and grow in milk, due to the lack of some nutritional ingredients in milk essential for the strains, which limits the research and application of fermented milk of *L. plantarum* (35, 36).

In this study, pregnant mice were exposed to VPA and offspring mice with autism-like behaviors were screened for the intervention experiments. Meanwhile, our team used our own patented technology (36) to ferment and curd *L. plantarum* ST-III directly in milk to get the fermented milk. We evaluated the effects of dietary changes and probiotic (*L. plantarum* ST-III-fermented milk) intake on the improvement of autism-like behavior and gut microbiota composition and explored the relationship between gut microbiota changes and ASD-related symptoms.

## Materials and methods

### Sample preparation

The preparation method of the intervention sample refers to our existing patent (WIPO patent WO2018049853A1) (36). Briefly, 80% raw milk, 2% proliferation agent, 7% sucrose, 5% orange juice and an appropriate amount of thickener were homogeneously mixed at 65°C and 20 MPa. After high temperature sterilization at 95°C for 10 min, it was cooled to 37°C and then inoculated with *L. plantarum* ST-III. The proliferation agent is a mixture produced by enzymatic hydrolysis reaction of soybean protein and pineapple whole juice at 25–80°C for 0.5–8 h, which could make *L. plantarum* ST-III strain grow faster and better in milk and increase the content of live bacteria. After incubation under 37°C for 12 h, the viable count was measured to be 5.0 × 10^8^ CFU/mL.

### Animals and experimental design

Healthy ICR mice of childbearing age were purchased from Shanghai Jiesijie Laboratory Animal Co., Ltd., and fed in a SPF-level laboratory animal room having a constant temperature of 24°C, relative humidity of 30–60% and alternating light of day and night for 12/12 h. After 1 week of adaptation, the male and the female (female: male = 2:1) mice were caged together overnight at 5 p.m., and the female mice were checked for the presence of vaginal plugs when they were separated at 9 a.m. the following day. The female mice having success fertilization were randomly divided into two groups, which were given a single intraperitoneal injection of VPA 500 mg/kg or equal volume of sterile saline solution on the 12.5 day of gestation. The offspring mice were subjected to behavioral tests from 6 to 8 weeks after birth. Compared with the control group, mice with obvious autistic-like behaviors were selected as ASD model mice for subsequent experiments. A total of 80 mice (20 normal control mice and 60 ASD model mice) were randomly divided into four groups, namely control group (normal control mice fed with sterile saline), VPA group (ASD model mice fed with sterile saline), ST-III group (ASD model mice fed with *L. plantarum* ST-III -fermented milk) and milk group (ASD model mice fed with milk containing proliferation agent), with 20 mice in each group, including 10 males and 10 females. All mice were given gavage administration of 400 μL solution in the morning and evening for 2 weeks, after which the behavioral test was performed. The mice were free to drink and eat during the intervention. All animal experimental procedures were carried out according to the governmental guidelines and approved by the Ethical Committee for Animal Research of the Shanghai Institute of Planned Parenthood Research (protocol code 2020-22, approval date 5/27/2020).

### Behavioral test

Behavioral tests were performed in the order of open field exploration and three chambers social test. At each test, the test mice were gently place back into the cage and the device was then wiped with 75% alcohol and allowed to dry for the next mouse test.

#### Open field test

The open field test is widely used to measure anxiety-like and locomotor behaviors in rodents. The test mice were placed in an open field of 50 cm × 50 cm × 40 cm and allowed to explore freely for 10 min to adapt to the environment. Then, the camera system (1/3″SONY Super HAD CCD) was turned on to record the total movement distance and central area residence time in the next 10 min for subsequent analysis.

#### Three chamber social test

**FIGURE 1:**
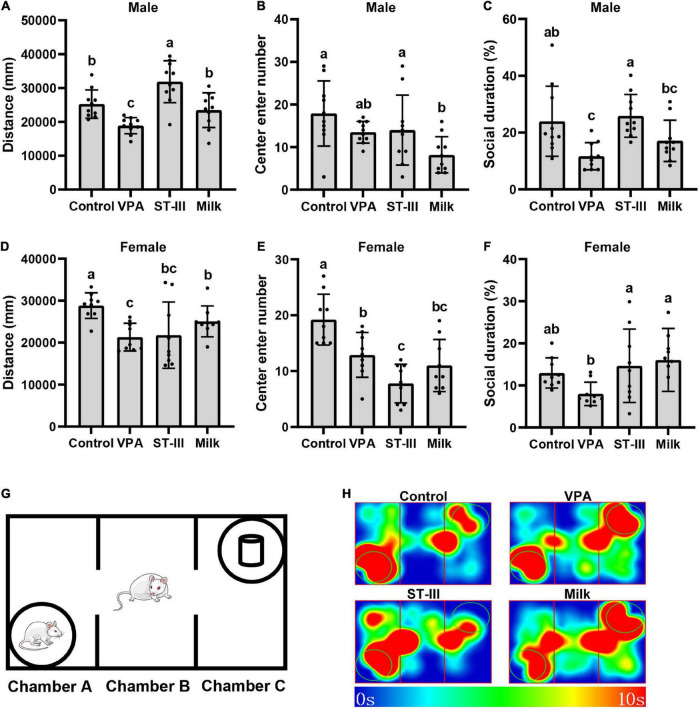
Valproic acid (VPA) offspring mice exhibit autistic-like behavior abnormality, and administration treatment of *Lactiplantibacillus plantarum* ST-III-fermented milk ameliorates the behavior deficits. **(A–C)** Total movement distance, number of entering central area and social duration of male mice. **(D–F)** Total movement distance, number of entering central area and social duration of female mice. **(G)** Schematic of the three-chamber social test. **(H)** Thermodynamic diagram of male mice in three chamber social test. All data are expressed as mean ± standard deviation, and differences between groups are compared by one-way analysis of variance (ANOVA) with LSD or Duncan *post-hoc* analysis to correct for multiple comparisons. Differences were considered statistically significant when the *p* < 0.05. **(a–c)** Different superscript letters indicate statistically significant differences, and same superscript letters indicate statistically insignificant differences. *n* (male) = 10 per group, *n* (female) = 9 per group.

Three chamber social test was used to evaluate social behaviors of mice. The test was conducted as previously study with some modifications. Briefly, a 60 cm × 40 cm × 22 cm plexiglass box was divided into three interconnected chambers (A–C). Mice were first habituated for 10 min to the full empty arena and then mice were confined in the B chamber (center chamber). A matched unfamiliar mouse with same genotype, age, sex and treatment was placed in the A chamber while a small object was placed in the C chamber (Figure 1G). For the next 10 min, the test mice were allowed to move freely in three chambers, and an overhead camera (1/3″SONY Super HAD CCD) recorded the olfactory interacting time between the tested mice and the strange mice.

### Sample collection and 16S rRNA gene sequence analysis

After 2 weeks of intervention, the mouse fecal samples were collected by taking individual mice out of cages and were immediately stored in −80°C until DNA extraction. The genomic DNA was extracted from 30 mg feces of mice using QIAamp DNA stool mini kit (Qiagen, Hilden, Germany) according to the protocol. A total of 1.5% agarose gel electrophoresis was used to check the integrity of extracted DNA. The 16S rRNA gene V3–V4 variable region was amplified by the primers 341F (5′-CCTACGGGNGGCWGCAG-3′) and 805R (5′-GACTACHVGGGTATCTAATCC-3′). The thermocycling steps were as followed: 95°C for 5 min, 20 cycles of at 95°C for 45 s, 55°C for 30 s, 72°C for 30 s and a final extension step at 72°C for 10 min. A 2% agarose gel was used for the recovery of the PCR products of each sample with the AxyPrep DNA Gel Extraction Kit (Axygen Inc., Union City, CA, USA) for the purification. All the amplicons were equivalently pooled after spectrophotometric assessment (QuantiFluor-ST; Promega, Madison, WI, USA), and sequenced according to Illumina MiSeq platform standard operating procedures.

### Bioinformatics analysis

The quality-controlled sequences were generated using DADA2 plugin of QIIME2 (version 2021.4) (37) from raw paired FASTQ files, then were clustered according to 97% similarity to obtain the representative OTU sequences using VSEARCH plugin. Community richness, evenness and diversity analysis (ACE, Shannoneven and Shannon) were performed using QIIME2 (version 2021.4) with the same sequence depth (24,797 sequences per sample). Principal Co-ordinates Analysis (PCOA) was performed using the Weight Unifrac algorithm at the OTU-level to assess species composition differences. Diversity of bacterial community compositions between groups was assessed using ANOSIM based on Bray-Curtis distance. Taxonomy was assigned using the online software RDP classifier at default parameter (80% threshold) based on the Ribosomal Database Project. To determine taxa significantly differential abundance in four groups, STAMP was applied to assess the abundance from phylum level to genus levels with *p*-value < 0.05 (38). Pair-wise Spearman correlation analysis was performed to explore co-occurrence patterns of microbial feature on genus level, and significant correlations (*p* < 0.05) were used in co-occurrence network construction by an open source software platform Cytoscape (version 3.2.1) (39). PICRUSt2 was used to infer the microbes’ metabolic functions based on the MetaCyc database (40). The coefficient relationship between genus and metabolic pathways was calculated using R package with Spearman correlation algorithm, and the correlation parameters were set as: coefficient >0.68 or <−0.68 and *p* < 0.05.

### Statistical analysis

SPSS22.0 software was used for data analysis, and GraphPad Prism 9 was used to make various statistical graphs. All data are expressed as mean ± standard deviation, and differences between groups are compared by one-way analysis of variance (ANOVA) with LSD or Duncan *post-hoc* analysis to correct for multiple comparisons. Differences were considered statistically significant when the *p* < 0.05.

## Results

### Administration of milk and *Lactiplantibacillus plantarum* ST-III-fermented milk alters valproic acid-induced autistic-like abnormal behaviors in mice

After 2 weeks of intervention, the behavior tests were performed. In the open field test, we recorded the total movement distance and the number of entering central area, which could reflect the locomotor behavior and anxious state. The results showed that compared with normal control group, the total movement distance of male and female mice in VPA group significantly decreased. In male mice, both the ST-III and milk group restored the locomotor behavior of mice, especially the total movement distance of the ST-III group was significantly higher than that of the control group. However, in female mice, comparing with the VPA group, the administration of *L. plantarum* ST-III-fermented milk seemed to have no effect; the milk group increased the total movement distance to a certain extent, but still did not return to normal levels (Figures 1A,D). The results of the number of entering central area showed that the VPA group in male mice had a decreasing trend without statistical significance, while female mice significantly decreased the entering number compared with control group. Both intervention of *L. plantarum* ST-III -fermented milk and milk seemed to be unable to significantly improve the anxiety-like behavior of either male or female mice, even had opposite effects (Figures 1B,E).

The three-chamber social test is used to measure the impairments in social interaction of mice. Male VPA offspring mice exhibited significant deficits in sociability since they preferred to interact with a novel object rather than a novel mouse. Treatment with *L. plantarum* ST-III -fermented milk significantly improved the social duration comparing with VPA group, while the effect of the milk treatment was not that obvious but still had an increasing trend (Figures 1C,H). VPA group in female mice showed a reduction trend of the proportion of social time (no statistical significance), and both ST-III and milk group significantly improved this trend comparing with the VPA group (Figure 1F).

Altogether, VPA-induced offspring mice showed autistic-like abnormal behaviors, including the decrease of locomotor behavior, anxiety and deficient sociability. Oral treatment with *L. plantarum* ST-III -fermented milk ameliorated ASD-related behavioral abnormalities in male mice but seemed not effective in female mice. Dietary changes caused by the administration of the mixture of milk and proliferation agent also seemed to have some positive or negative effects on autism-like behaviors.

According to the behavioral test results, we found that after the intervention of *L. plantarum* ST-III fermented milk, male mice showed more obvious improvement in VPA-induced autism-like behavior comparing with female mice. Therefore, in the following study, we focused on the effect of dietary changes and probiotic intake on the gut microbiota of male mice.

### Administration of milk and *Lactiplantibacillus plantarum* ST-III-fermented milk increases the gut microbiota diversity in male valproic acid mice

**FIGURE 2:**
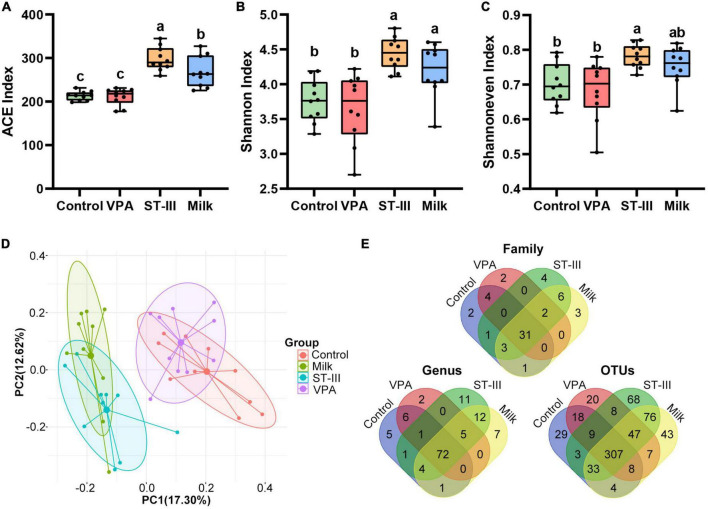
The effect of milk and *Lactiplantibacillus plantarum* ST- III -fermented milk on gut microbial diversity in male VPA mice. **(A–C)** Alpha-diversity in terms of the ACE, Shannoneven and Shannon index. **(D)** Bray-Curtis distance-based PCoA plot of all OTUs from feces of control, VPA, ST-III and milk group. **(E)** Venn diagram at family, genus and OUT levels. All data are expressed as mean ± standard deviation, and differences between groups are compared by one-way analysis of variance (ANOVA) with LSD or Duncan *post- hoc* analysis to correct for multiple comparisons. Differences were considered statistically significant when the *p* < 0.05. **(a–c)** Different superscript letters indicate statistically significant differences, and same superscript letters indicate statistically insignificant differences. *n* = 10 per group.

The alpha diversity analysis of male mice showed that no statistical difference were observed in the ACE index (richness), Shannoneven index (evenness), and Shannon index (structure) of gut microbiota between the control group and VPA group, while both the ST-III group and milk group exhibited a significant increase (*p* < 0.05) or an increasing trend relative to the control group and VPA group (Figures 2A–C), indicating that the abundance, evenness and diversity of gut microbiota in the intervention group (ST-III group + milk group) were higher than those of non-intervention group (control group + VPA group). Notably, the ST-III group had the highest ACE index among the four groups (Figure 2A).

The PCoA analysis revealed treatment-related clustering, with a marked difference between the intervention group (ST-III group + milk group) and non-intervention group (control group + VPA group) (ANOSIM, *R* = 0.6294, *p* < 0.001) (Figure 2D), indicating that 2 weeks of milk and *L. plantarum* ST-III -fermented milk treatment could change the gut microbiota of mice. ANOSIM analysis also supported that there is no significant difference between the control group and VPA group (*R* = 0.1134, *p* = 0.066), or the ST-III group and milk group (*R* = 0.1762, *p* = 0.022).

As shown in Figure 2E, at the family, genus and OTU levels, the common number in these four groups was 31, 72, and 307, respectively; and the ST-III group contained the highest number of unique microbes, followed by the milk group. In contrast, the VPA group had the lowest number of unique microbes at the genus and OTU levels. These findings indicated that dietary changes and probiotic intake significantly influenced the alpha diversity of gut microbiota in male offspring mice, whereas maternal injection of VPA had little effect. Moreover, consuming fermented milk with *L. plantarum* ST-III was more beneficial for increasing the diversity of gut microbiota.

### Administration of milk and *Lactiplantibacillus plantarum* ST-III-fermented milk alters the gut microbiota composition in male valproic acid mice

**FIGURE 3:**
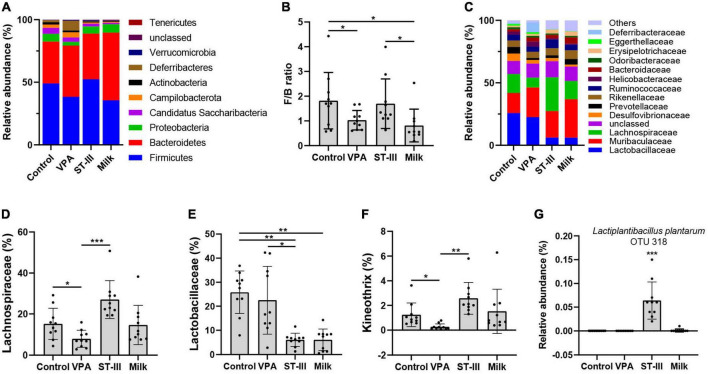
The effect of milk and *Lactiplantibacillus plantarum* ST- III -fermented milk on gut microbial composition in male VPA mice. **(A)** Phylum level. **(B)** *Firmicutes*/*Bacteroidetes* (F/B) ratio. **(C)** Family level. Relative abundance of **(D)** *Lachnospiraceae*, **(E)** *Lactobacillaceae*, **(F)** *Kineothrix*, and **(G)** *L. plantarum* in control, VPA, ST-III and milk group. *n* = 10 per group. *p < 0.05, **p < 0.01, ***p < 0.001.

Analysis of changes in the gut microbiota composition of fecal samples from male mice at the level of phylum is presented in Figures 3A,B and Table 1. *Firmicutes* and *Bacteroidetes* were the dominant bacteria in the gut microbiota (82.43 ± 3.13%, 79.26 ± 15.20%, 88.92 ± 3.85%, and 89.61 ± 9.03% in the control, VPA, ST-III and milk group, respectively). The relative abundance of *Firmicutes* in the VPA (38.40 ± 8.91%) and milk (35.50 ± 13.18%) group showed a significant decrease comparing with the control group (49.01 ± 12.00%) (*p* < 0.05 and *p* < 0.01, respectively), while recovered to 52.36 ± 8.75% in the ST-III group (*p* < 0.01) (Figure 3A and Table 1). The *Firmicutes*/*Bacteroidetes* (F/B) ratio was 1.82 ± 1.14 in the control group and decreased to 1.03 ± 0.39 (*p* < 0.05) and 0.81 ± 0.66 (*p* < 0.05) in the VPA and milk groups, respectively, while the ratio in the ST-III group was 1.70 ± 1.00, closing to the control group (Figure 3B and Table 1). We also found that the relative abundance of *Deferribacteres* in the VPA group increased from 1.64 ± 2.41% (control group) to 7.86 ± 14.93% (no statistical difference), which was mainly attributed to the increase of species *Mucispirillum schaedleri*. After the intervention of *L. plantarum* ST-III fermented milk and milk for 2 weeks, the level of *Deferribacteres* decreased to 0.41 ± 0.48% and 0.16 ± 013% (no statistical difference), respectively (Table 1).

**TABLE 1:** Relative abundance of gut microbiota and F/B ratio in the control, VPA, ST-III and milk group at the level of phylum.

Phylum	Control group	VPA group	ST-III group	Milk group
*Firmicutes*	49.01 ± 12.00%	38.40 ± 8.91%*	52.36±8.75%##	35.50 ± 13.18%**ΔΔ
*Bacteroidetes*	33.41 ± 12.55%	40.86 ± 11.43%	36.56 ± 11.43%	54.11 ± 16.68%**^#^ΔΔ
*Firmicutes* + *Bacteroidetes*	82.43 ± 3.13%	79.26 ± 15.20%	88.92 ± 3.85%^#^	89.61 ± 9.03%^#^
*Firmicutes*/*Bacteroidetes*	1.82 ± 1.14	1.03 ± 0.39*	1.70 ± 1.00	0.81 ± 0.66*Δ
*Deferribacteres*	1.64 ± 2.41%	7.86 ± 14.93%	0.41 ± 0.48%	0.16 ± 0.13%

All data are expressed as mean ± standard deviation, and differences between groups are compared by one-way analysis of variance (ANOVA) with LSD or Games-Howell analysis to correct for multiple comparisons. *n* = 10 per group. *p < 0.05 and **p < 0.01 compared with the control group. ^#^*p* < 0.05 and ^##^*p* < 0.01 compared with the VPA group. Δ*p* < 0.05 and ΔΔ*p* < 0.01 compared with the ST-III group.

At the family level, we could observe that the dominant bacteria of mice gut microbiota in the intervention group and the non-intervention group changed significantly (Figure 3C). The changes in the composition of the gut microbiota in the four groups were mainly distributed in *Lactobacillaceae*, *Lachnospiraceae*, and *Desulfovibrionaceae* (Figure 3C and Supplementary Figure 1). The relative abundance of *Lachnospiraceae* was significantly decreased in the VPA group relative to the control group (*p* < 0.05). We found that the administration of *L. plantarum* ST-III, not the milk, markedly restored the relative abundance of *Lachnospiraceae* (*p* < 0.001) (Figure 3D). Besides, we also found that the relative abundance of *Lactobacillaceae* was significantly lower in the intervention groups (ST-III group + milk group) compared with the control group (*p* < 0.01 and *p* < 0.01, respectively) (Figure 3E).

At the genus level, the relative abundance of *Kineothrix* was reduced in the VPA group (*p* < 0.05) compared with the control group, while a significant increase was detected in ST-III group compared with the VPA group (*p* < 0.01) (Figure 3F). In addition, *Desulfovibrio* and *Adlercreutzia* in both ST-III and milk groups were decreased compared to the control group (*p* < 0.05); *Acetatifactor* in ST-III group was significantly increased compared with the VPA group (*p* < 0.05); *Lachnospira* was significantly increased in the milk group relative to the control and VPA group (*p* < 0.05) (Supplementary Figure 1). Remarkably, our sequencing results also showed that species *L. plantarum* (OTU318) was almost absent in the other three groups and was detected only in ST-III group (Figure 3G), indicating that *L. plantarum* ST-III was able to be resistant to gastric acid, and persisted in the intestinal tract of mice with certain functional activities.

### Co-occurrence between *Lactiplantibacillus plantarum* ST-III and intestinal microbiota of male valproic acid mice

**FIGURE 4:**
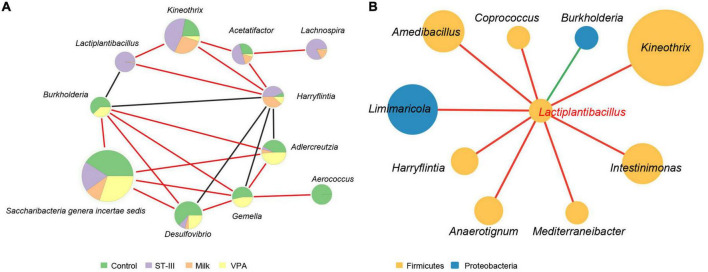
The biological network of gut microbiota in mice. **(A)** Network analysis showed co-occurrence among gut microbes in mice. Each node represents a genus of bacteria, and the size of the node is proportional to the relative abundance of that organism in the microbial community. The different color in the circles indicate the proportion of the relative abundance of the genus among the four groups. Red and black lines between nodes imply a positive and negative relationship between bacteria, respectively. **(B)** Network analysis showed co-occurrence between *Lactiplantibacillus* and other gut microbes in mice. Each node represents a genus of bacteria with the color indicating the phylum to which the genus belongs. The size of the node is proportional to the relative abundance of that organism in the microbial community. Red and green lines between nodes imply a positive and negative relationship between bacteria, respectively. Only taxa with correlation significance less than 0.05 are shown.

Network analysis was used to explore co-occurrence of bacteria that was significantly affected (*p* < 0.05) by diet and administration of *L. plantarum* ST-III. As shown in Figure 4A, *Harryflintia* was positively correlated with *Kineothrix* and *Acetatifactor*, and negatively associated with *Burkholderia*, *Desulfovibrio*, *Gemella*, and *Adlercreutzia*. On the contrary, *Burkholderia* was found to be positively correlated with *Saccharibacteria genera incertae sedis*, *Desulfovibrio*, *Gemella*, and *Adlercreutzia*. Notably, *Lactiplantibacillus* (predominantly present in the ST-III group) was found to be well integrated into the co-occurrence networks, with not only a significant positive correlation with *Kineothrix* and *Harryflintia*, but also a significant negative correlation with *Burkholderia*.

We next focused on the co-occurrence between *Lactiplantibacillus* and other intestinal microbes (Figure 4B). We found that in addition to the three genera mentioned above (*Kineothrix*, *Harryflintia*, and *Burkholderia*), *Lactiplantibacillus* was positively correlated with *Coprococcus*, *Intestinimonas*, and *Mediterraneibacter*, which were capable of producing short-chain fatty acid (butyrate) (41, 42).

### Functional features of the intestinal microbiota for male valproic acid mice

**FIGURE 5:**
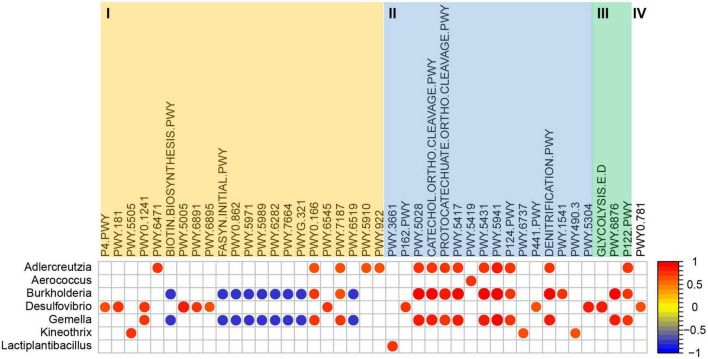
Coefficient relationship between differential bacteria (genus level) and metabolic pathways analyzed using PICRUSt2 based on the 16S rRNA amplicon data. Only results with *p* < 0.05 and correlation coefficient >0.68 or < –0.68 are shown in the figure. I = Biosynthesis; II = Degradation/Utilization/Assimilation; III = Generation of precursor metabolites and energy; IV = Superpathways.

The functional profiling assessment through PICRUSt2 produced a total of 361 metabolic pathways in microbial populations. Compared with the control group, the abundance of 65 metabolic pathways was significantly changed in VPA induced mice model, but 25 of them showed significantly opposite change after *L. plantarum* ST-III intervention, including butanoate production and glutamine biosynthesis (Supplementary Table 1). To predict the association of these metabolic pathways with significantly enriched or diminished bacteria (genus) due to *L. plantarum* ST-III supplementation, Spearman correlation analysis was performed. As shown in Figure 5, 75 pathways of four functional categories (biosynthesis, degradation/utilization/assimilation, generation of precursor metabolites and energy, and superpathways) showed high correlations (*R* > 0.68 or < −0.68) with seven significantly changed genera (Supplementary Table 2). Genus *Lactiplantibacillus* and *Kineothrix* were enriched in the ST-III group. *Lactiplantibacillus* was positively correlated with one pathway related to amine and polyamine degradation; *Kineothrix* was positively correlated with three pathways related to amino acid biosynthesis, carbohydrate degradation, and inorganic nutrient metabolism. *Burkholderia* had a direct co-exclusion relationship with *Lactiplantibacillus*, while *Adlercreutzia* and *Gemella* had indirect co-exclusion relationship (Figure 4), and they were all mainly enriched in the control and VPA groups. These three genus were positively correlated with 11 pathways related to nucleoside and nucleotide biosynthesis, amino acid degradation, aromatic compound degradation and carbohydrate degradation. Among them, *Burkholderia* and *Gemella* were also negatively correlated with nine pathways related to cofactor, carrier, and vitamin biosynthesis, fatty acid and lipid biosynthesis, and other biosynthesis.

## Discussion

This study aimed to evaluate the effects of dietary changes and probiotic (*L. plantarum* ST-III -fermented milk) intake on ASD mouse model. ASD is a complex developmental disability with unclear etiology and no effective or targeted cure (25). Researchers have developed a variety of animal models to explore its pathogenesis and treatment methods, such as zebrafish, monkeys, and songbirds, but the main experimental models are rodents (43). Here, we injected 500 mg/kg of VPA, a drug for epilepsy and mood swings (9) and an environmental risk factor during pregnancy associated with developing ASD in offspring, on day 12.5 of pregnancy. Compared with the control group, male offspring of the VPA group showed significant abnormalities in the total movement distance and the three chambers social test, while there was a decreasing trend in the number of entering central area. On the other side, female VPA offspring mice showed a significant decrease in locomotor behavior and a significant increase in anxiety behavior, but not significant abnormalities in sociability. In mammals, both the prenatal and postnatal periods were the key developmental windows that ultimately affect adult behaviors (44). Kim et al. (45) have reported that exposure to VPA on day 12 of gestation in maternal rodents was ideal as the offspring showed significant behavioral deficits and no other significant teratogenicity (such as tail deformities). In addition, as an environmental factor induced model mice, the VPA-induced ASD mice are usually used for the study of the gut microbiota-ASD relationship (46). These studies have shown that rodent models with prenatal VPA exposure were useful for ASD animal model.

Another noteworthy phenomenon was the gender specificity in VPA-induced behavioral abnormalities, characterized by sociability deficits in male mice and anxiety symptoms in female mice. Schneider et al. (47) evaluated rodent VPA ASD models using a series of behavioral, immunological, and endocrinological tests, and found that male offspring rats had more behavioral abnormalities than the female ones. It was also found in another study that prenatal VPA exposure increased excitatory neuron differentiation and decreased inhibitory neuron differentiation in the offspring of both male and female rats, but postsynaptic changes in the cortex were mainly limited to the male ones, indicating that ASD rat models showed a male preference for decreased inhibitory function in the brain (45). A clinical study of twin samples from TEDS (Twins Early Development Study) and CATSS (Child and Adolescent Twin Study of Sweden) showed females with ASD are protected against some of the symptoms of ASD (often called the female protective effect or FPE) (46).

The Food and Agriculture Organization and the World Health Organization define probiotics as living organisms which, when administered in adequate amounts, confer a health benefit to the host. While the beneficial effects of probiotics on gastrointestinal function are generally well recognized, a growing body of new studies have suggested potential therapeutic benefits of probiotics in several central nervous system (CNS) conditions through the “Gut-Microbiota-Brain Axis,” such as anxiety, depression, ASDs and Parkinson (48). *L. plantarum* has been shown to have multiple benefits for human health, including promoting gut integrity and motility, altering intestinal microbiota and inhibiting growth of potential pathogens, modulating human immune function, and the ability to treat and ameliorate human disease (26). In this study, we evaluated the effect of *L. plantarum* ST-III-fermented milk improving behavioral deficits in VPA-induced ASD mice. After the administration of *L. plantarum* ST-III-fermented milk to ASD mice for 2 weeks, the behavioral performance was observed. We found that probiotic intervention, but not milk, significantly improved locomotor behavior and sociability in male ASD mice. In contrast, probiotics intervention seemed to be less effective in improving behavioral deficits in female ASD mice. Specifically, ST-III group was not as effective as the milk group in the improvement of total movement distance and social duration, and even had the opposite effect in experiments reflecting anxiety behavior in female mice.

This kind of differences in treatment effect associated with gender and behavior were widely reported. Hsiao et al. (25) used maternal immune activation (MIA) mouse as an autism model and have found in their studies that oral administration of the gut commensal *Bacteroides fragilis* could improve the integrity of gut barrier and ameliorated atypical anxiety, communicative, repetitive, and sensorimotor behavioral symptoms, but not sociability or social preference, in the polyinosinic-polycytidylic acid-induced ASD mice. Sgritta et al. (44) reported that treatment of *Lactobacillus reuteri* MM4-1A acted in a vagus nerve-dependent manner and selectively rescued social deficits in three types of male ASD model mice, including the VPA environmental model, the BTBR idiopathic model and the *Shank3B*^–/–^ model. In a clinical study, the effects of *L. plantarum* PS128 on boys with ASD was investigated by giving PS128 capsules to 36 children for 4 weeks. The results showed that PS128 ameliorated anxiety behavior, and decreased hyperactivity and opposition/defiance behaviors (34). Data from rodent models and preliminary clinical studies provide arguments on the potential effect of probiotic treatments on ASD-related behavioral deficits, and on the complex interplay of the gut microbiota and nervous system in ASD. Each strain or even species of probiotic could have a different influence on ASD symptoms, and our data suggested that *L. plantarum* ST-III could effectively ameliorate VPA-induced autism-like behaviors, especially sociability deficits in male ASD mice.

In the “Gut-Microbiota-Brain Axis” theory, gut microbes play an important role in two-way communication system between the gut and the brain (49). Many studies have reported alterations in gut microbiota in ASD (25). In animal experiments, VPA rat model of ASD exhibited altered gut microbial composition and reduced richness in a pattern similar to that seen in patients with ASD (50). Generally, a higher diversity of gut microbiota is considered to be a healthier gut environment, as the integrated gut microbiota is able to protect gut from environmental risk factors (51). Therefore, we studied the gut microbiota of male ASD mouse models using 16S rRNA gene sequencing to survey the changes associated with probiotic intervention. Interestingly, in contrast to other studies, our results revealed an increased alpha diversity of gut microbiota in the intervention group (especially in the ST-III group), and no significant differences between VPA-exposed and control mice. The finding of de Theije et al. was consistent with our results (52). They investigated microbiota composition in mice *in utero* exposed to VPA and found that the microbial diversity and species richness showed no differences between ASD mice and control mice. The PCoA analysis also showed a markedly altered microbial community structure between the intervention and non-intervention groups. The VPA group had the least number of unique microbiota, while the ST-III group had the most. Thus, we suggested that the dietary shifts in the intervention group were an important determinant of increased gut microbial diversity in mice, and the intake of *L. plantarum* ST-III-fermented milk had a more significant effect than the milk group without probiotics.

Besides the microbiota diversity, several key differentiating bacterial taxa with significant differences between the four groups were identified. The ratio of F/B, which is tightly related to ASD and obesity (53), decreased in the VPA and milk group comparing with the control group, and returned to normal levels in the ST-III group. The relative abundance of *Deferribacteres* was observed to be elevated in the VPA group, and further analysis revealed that the increase of *M. schaedleri* was the main cause of this change. *M. schaedleri* is a gram negative and obligate anaerobic bacterium, which is a dominant member of the cecal crypt microbiome in mice and was found to be sufficiently invasive to prime adaptive immune responses in a normal host context (54). In the ST-III group and the milk group, the trend of increasing the abundance of *Deferribacteres* was suppressed. At the family level, the relative abundance of *Lachnospiraceae*, which was significantly reduced in the VPA group, was found to increase significantly after *L. plantarum* ST-III-fermented milk intake, but there was no such improvement in the milk group. *Lachnospiraceae* is an abundant family of anaerobic bacteria in healthy humans and impact their hosts by converting primary bile acids to secondary bile acids, producing short-chain fatty acids or lantibiotics, an important class of peptide antibiotics, and facilitating colonization resistance against intestinal pathogens. A decrease in *Lachnospiraceae* abundance may have negative health effects (55–57). Another family that showed significant changes after intervention was *Lactobacillaceae*. We found that the intake of skim milk (with proliferation agent) and ST-III fermented milk decreased the abundance of *Lactobacillaceae*, which was mainly attributed to the reduction of genus *Lactobacillus* (from 13.9% (Control group) and 15.01% (VPA group) to 3.19% (ST-III group) and 3.27% (Milk group), respectively). The prior finding of Yin et al. showed that the relative abundance of ingested *L. plantarum* WCFS1 was significantly increased during mouse consumption of HFHSD (high-fat, high-sugar diet) and was negatively associated with the numbers of indigenous *Lactobacillus* in mice intestines (58). Reduced *Lactobacillus* abundance was also found in other rodent studies with altered diets (14). At the genus level, the abnormal reduction of *Kineothrix* induced by VPA exposure was significantly improved in the ST-III group. Individual studies have reported significantly lower intestinal *Kineothrix* levels in both Parkinson’s patients and constipation model mice (59). Liddicoat et al. found that an increase in the relative abundance of *Kineothrix alysoides*, a butyrate-producing anaerobic species, was associated with the decrease in anxiety-like behavior in mice (60). In addition, compared with the VPA group, the abundance of *Acetatifactor* was significantly increased in the ST-III group, which produces acetate and butyrate (61). Therefore, we supposed that the macronutrients alteration caused by diet seemed to be an important factor affecting the composition of the indigenous microbiota in mice, while additional probiotic intake (*L. plantarum* ST-III and its metabolites) was able to modulate the abundance of specific microbes in the gut of ASD mice.

To determine whether *L. plantarum* ST-III has a direct or indirect effect on the structure of the gut microbiota in ASD mice, we performed the network analysis to explore co-occurrence among the microbiota. *Lactiplantibacillus* (predominantly present in the ST-III group) was found to be well integrated into the co-occurrence network and had direct co-occurrence or co-exclusion relationship with *Kineothrix*, *Harryflintia*, and *Burkholderia*. *Harryflintia*, a genus of the family *Ruminococcaceae*, has been found contributing to the antidepressant effect (62). Genus *Burkholderia* includes a wide variety of bacterial species that are pathogenic to humans and other vertebrates. *Burkholderia* is associated with chronic infections, such as pneumonia and gastrointestinal infections, and is intrinsically resistant to major classes of antibiotics (63). The genera that were negatively correlated with *Harryflintia* and positively correlated with *Burkholderia* included *Desulfovibrio*, *Gemella*, and *Adlercreutzia*. *Gemella* is an opportunistic pathogen that can cause life-threatening infections in individuals with risk factors (64). *Adlercreutzia* has been reported to be associated with depression in animal experiments (65). Our results indicated that *Harryflintia* and *Burkholderia* may play a role in alteration of the gut microbial composition, while *Lactiplantibacillus* may affect VPA mice gut microbiota by altering specific microbiota.

Additionally, a PICRUSt2 analysis was performed to infer the functional enrichment or reduction in the microbiome community. The abundance of 25 pathways were significantly changed (19 reduced and 6 increased) in VPA-induced ASD mice model, but the change was reversed after *L. plantarum* ST-III intervention (Supplementary Table 1). The pathway acetyl-CoA fermentation to butanoate II and L-glutamate and L-glutamine biosynthesis were both reduced in VPA-induced ASD mice model, and increased after *L. plantarum* ST-III intervention. Butanoate (Butyrate), as a short-chain fatty acid (SCFA), could attenuate social behavior deficits (66). The level of glutamine is decreased in children with ASD (67). So the abundance increase of the two pathways after *L. plantarum* ST-III intervention might be associated with autistic-like behaviors improvement. In the ST-III group, the enrichment of genus *Lactiplantibacillus* was positively correlated with the functional pathway of amine and polyamine degradation, including the functional ID of PWY-3661 (glycine betaine degradation I). The enrichment of genus *Kineothrix* was positively correlated with the functional pathway of amino acid biosynthesis, carbohydrate degradation and inorganic nutrient metabolism, including PWY-5505 (L-glutamate and L-glutamine biosynthesis), PWY-6737 (starch degradation V) and PWY490-3 [nitrate reduction VI (assimilatory)]. The related functional pathways of *Burkholderia* and *Gemella* were mainly negatively correlated with the fatty acid and lipid biosynthesis pathway, and positively correlated with the aromatic compound degradation, carbohydrate degradation and nucleoside and nucleotide biosynthesis pathways. From the PICRUSt2 analysis, we speculated that the intervention of *L. plantarum* ST-III may alter the autism-related gut ecological environment and microbiota structure through enrichment of related functional pathways including PWY-3661, PWY-5505, PWY-6737, and PWY490-3.

## Conclusion

In this study, *L. plantarum* ST-III-fermented milk, not the milk alone, was effective in ameliorating the impaired social interaction and total movement distance in male ASD mice, but not the anxiety behavior exhibited by female mice. Meanwhile, it was found that dietary changes were the main cause of the altered gut microbiota in mice, and additional intake of probiotic *L. plantarum* ST-III-fermented milk seemed to improve autistic-like behaviors in male mice by modulating specific gut microbes, like increasing the relative abundance of family *Lachnospiraceae* and genus *Kineothrix*, which were significantly reduced in the VPA-induced ASD mice model. In addition, 25 metabolic pathways which were significantly changed in VPA-induced mice but reversed after *L. plantarum* ST-III intervention, like butanoate production and glutamine biosynthesis, might contribute to improving the autistic-like behaviors. In short, these findings have provided some experimental data for gut microbiota-based therapeutic intervention for ASD, suggesting that the probiotic fermented milk by *L. plantarum* ST-III may play a beneficial role in improving the behavioral symptoms of ASD, and is expected to be one of the candidate functional foods.

## Data availability statement

The datasets presented in this study can be found in online repositories. The names of the repository/repositories and accession number(s) can be found below: https://www.ncbi.nlm.nih.gov/genbank/, PRJNA860734.

## Ethics statement

The animal study was reviewed and approved by the Ethical Committee for Animal Research of the Shanghai Institute of Planned Parenthood Research.

## Author contributions

CY, HuZ, and ZL designed the experiments and reviewed and revised the manuscript before submission. MG, YZ, and RL performed the animal experiments and behavior experiments. HoZ performed the preparation of *L. plantarum* ST-III-fermented milk. MG and YW were responsible for the arrangement and analysis of data. YZ participated in interpreting the results and wrote the manuscript. All authors approved the final version of the manuscript.

## Supplementary material

Supplementary Figure 1 Relative abundance of the gut microbiota at the family (*f*) and genus (*g*) levels. **(A)** VPA group vs. Control group. **(B)** ST-III group vs. Control group. **(C)** Milk group vs. Control group. **(D)** ST-III group vs. VPA group. **(E)** Milk group vs. VPA group. **(F)** Milk group vs. ST-III group. *n* = 10 per group. **p* < 0.05, ^**^*p* < 0.01, and ^***^*p* < 0.001.
