# Supplementary figures and images for: Lactiplantibacillus plantarum ST-III-fermented milk improves autistic-like behaviors in valproic acid-induced autism spectrum disorder mice by altering gut microbiota
Source: Front Nutr. 2022 Nov 24;9:1005308. doi: 10.3389/fnut.2022.1005308 (PMC9729765; doi:10.3389/fnut.2022.1005308)

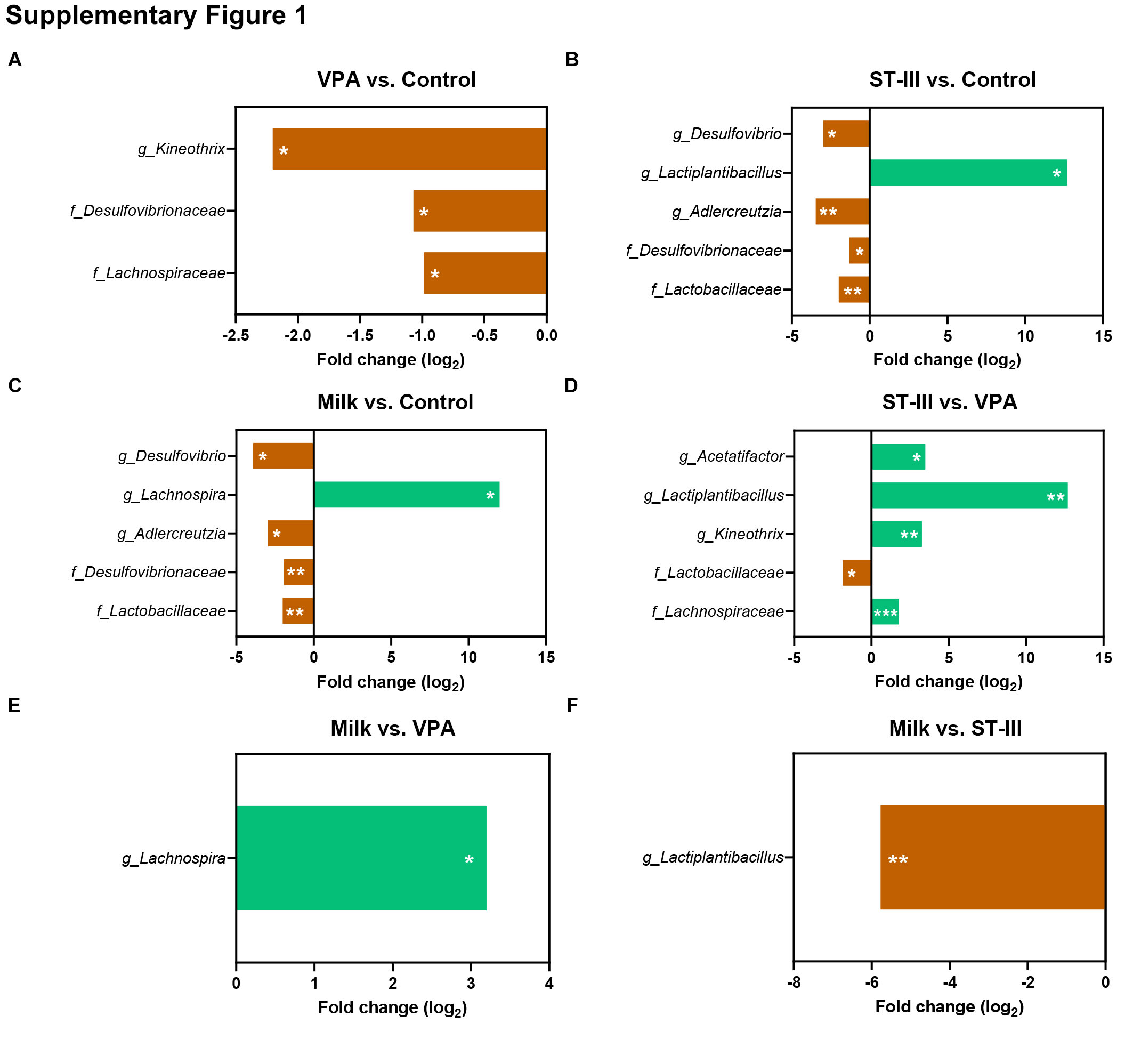

Supplement: Supplementary Figure 1 — Relative abundance of the gut microbiota at the family (f) and genus (g) levels. (A) VPA group vs. Control group. (B) ST-III group vs. Control group. (C) Milk group vs. Control group. (D) ST-III group vs. VPA group. (E) Milk group vs. VPA group. (F) Milk group vs. ST-III group. n = 10 per group. *p < 0.05, **p < 0.01, and ***p < 0.001. [file Image_1.JPEG]
